# Draft Genome Sequence of *Serratia fonticola* UTAD54, a Carbapenem-Resistant Strain Isolated from Drinking Water

**DOI:** 10.1128/genomeA.00970-13

**Published:** 2013-11-27

**Authors:** Isabel Henriques, Rommel Thiago Jucá Ramos, Rafael Azevedo Baraúna, Pablo Henrique de Sá, Diogo Marinho Almeida, Adriana Ribeiro Carneiro, Silvanira Barbosa, Anabela Pereira, Artur Alves, Maria José Saavedra, Conceição Egas, Artur Silva, António Correia

**Affiliations:** Department of Biology and CESAM, University of Aveiro, Campus Universitário de Santiago, Aveiro, Portugala; Instituto de Ciências Biológicas, Universidade Federal do Pará, Belém, Pará, Brazilb; Biocant-Biotechnology Innovation Center, Cantanhede, Portugalc; Department of Veterinary Sciences, CECAV, University of Trás-os-Montes e Alto Douro, Vila Real, Portugald

## Abstract

*Serratia fonticola* UTAD54 is an environmental isolate that is resistant to carbapenems due to the presence of a class A carbapenemase and a metallo-β-lactamase that are unique to this strain. Its draft genome sequence was obtained to clarify the molecular basis of its carbapenem resistance and identify the genomic context of its carbapenem resistance determinants.

## GENOME ANNOUNCEMENT

*Serratia fonticola* is a member of the family *Enterobacteriaceae* that occurs naturally in aquatic environments and that occasionally causes infections in humans (1). Members of this species express both a chromosomally encoded class A β-lactamase and an AmpC β-lactamase (2), and they are resistant to most β-lactam antibiotics except for carbapenems.

*S. fonticola* strain UTAD54 was isolated from a drinking water fountain in Portugal in 1998 (2). Its high level of resistance to carbapenems justified deeper characterization of the strain. According to the 16S rRNA gene sequence, *S. fonticola* UTAD54 is closely related to the type strain *S. fonticola* LMG 7882. However, *S. fonticola* UTAD54 is an exceptional strain in the sense that besides the naturally occurring β-lactamases, it produces two different carbapenemases: the class A β-lactamase SFC-1 (3, 4) and the metallo-β-lactamase Sfh-I (2, 5). These enzymes are not present in other *S. fonticola* strains, and the sequences of the genes (2, 3) and the kinetic properties (4, 5) and three-dimensional (3D) structures (6, 7) of the enzymes demonstrate that they are unique in the context of the structural and functional diversity of β-lactamases. Genes encoding these enzymes have been demonstrated to be located in the chromosome (3). Even so, these exceptional characteristics might be the result of the acquisition of genetic elements by horizontal gene transfer. We report here the draft genome sequence of *S. fonticola* UTAD54.

The genome sequence was determined using a Roche FLX 454 genome sequencer. A total of 104,697,796 bp was produced, and sequences were extracted from .sff file with the script “sff_extract_0_2_13” (http://bioinf.comav.upv.es/sff_extract/). Low-quality reads were removed using Quality Assessment Long Reads (8) using the average Phred quality of 20. The analysis resulted in 347,637 reads (104,043,448 bp), representing a coverage of ~18×.

*De novo* assembly was performed using the software Mira (9) and produced 141 contigs. SeqMan NGen version 11 was used to remove redundant sequences, curate alignments, and extend the sequences based on similarities in their ends. The draft genome sequence was distributed in 133 contigs, with an N_50_ length of 101 kb. The total size was estimated to be 5,953,423 bp, with a G+C content of 54%. The genome was annotated using the RAST server (10). A total of 5,349 coding sequences (CDSs), 71 tRNAs, and 4 rRNAs were predicted in the genome.

The emergence of carbapenem resistance in *Enterobacteriaceae* is a worldwide public health threat. The draft genome sequence of *S. fonticola* UTAD54 will enable the investigation of the molecular basis of carbapenem resistance in this strain as well as the genomic context of *bla*_SFC-1_ and *bla*_Sfh-I_. A comparative analysis with other *Serratia* spp., including the *S. fonticola* type strain, will be conducted in the near future in order to elucidate the processes that gave rise to a strain that represents a risk to humans in a genomic background of a nonpathogenic species.

### Nucleotide sequence accession numbers.

The *Serratia fonticola* UTAD54 draft genome sequence has been deposited in GenBank under the accession no. AUZV00000000. The version described in this paper is version AUZV01000000.
